# Pancreatic cancer pathology viewed in the light of evolution

**DOI:** 10.1007/s10555-020-09953-z

**Published:** 2021-02-08

**Authors:** Michaël Noë, Seung-Mo Hong, Laura D. Wood, Elizabeth D. Thompson, Nicholas J. Roberts, Michael G. Goggins, Alison P. Klein, James R. Eshleman, Scott E. Kern, Ralph H. Hruban

**Affiliations:** 1 Sol Goldman Pancreatic Cancer Research Center, Department of Pathology, The Johns Hopkins University School of Medicine, Carnegie 415, 600 North Wolfe Street, Baltimore, MD 21287, USA; 2 Sol Goldman Pancreatic Cancer Research Center, Department of Oncology, The Johns Hopkins University School of Medicine, Baltimore, MD 21287, USA; 3 Department of Pathology, Asan Medical Center, University of Ulsan College of Medicine, Seoul, Republic of Korea; 4 Sol Goldman Pancreatic Cancer Research Center, Department of Medicine, The Johns Hopkins University School of Medicine, Baltimore, MD 21287, USA; 5 Department of Epidemiology, Bloomberg School of Public Health, The Johns Hopkins University School of Medicine, Baltimore, MD 21231, USA

**Keywords:** Pancreas cancer, Pancreatic cancer, Darwin, Evolution, Pancreatic intraepithelial neoplasia, Intraductal papillary mucinous neoplasm

## Abstract

One way to understand ductal adenocarcinoma of the pancreas (pancreatic cancer) is to view it as unimaginably large numbers of evolving living organisms interacting with their environment. This “evolutionary view” creates both expected and surprising perspectives in all stages of neoplastic progression. Advances in the field will require greater attention to this critical evolutionary prospective.

## 1 Introduction

Theodosius Dobzhansky famously wrote that “nothing in the biological aspects of medicine makes sense except in the light of evolution,” and yet, with the exception of elegant mathematical modeling based on evolutionary principles, much of today’s discourse on pancreatic cancer fails to include an evolutionary perspective [1–4]. Here we ask if any insights can be gained by viewing the pathology and genetics of pancreatic cancer in the light of evolution, and we find several instances in which doing so reveals new understandings of the disease.

To set the stage for looking at pancreatic cancer in the light of evolution, we thought it would be helpful to first consider some of Darwin’s own words. In the *Origins of Species By Means of Natural Selection*, Darwin wrote:

As many more individuals of each species are born than can possibly survive; and as, consequently, there is a frequently recurring struggle for existence, it follows that any being, if it vary however slightly in any manner profitable to itself, under the complex and sometimes varying conditions of life, will have a better chance of surviving, and thus be *naturally selected*. From the strong principle of inheritance, any selected variety will tend to propagate its new and modified form [5].

This first passage succinctly encompasses the fundamental drivers through which we will examine pathology and genetics of infiltrating ductal adenocarcinoma (referred to in this review simply as “pancreatic cancer”). These evolutionary drivers include the following: (1) that more organisms are born than can survive; (2) that they compete for limited resources; (3) that the environment in which organisms live changes; and (4) that those that are best suited for their changing environment are more likely to survive. We are referring to these principles when we use the term “evolution” in this review.

These evolutionary drivers influence the development of pancreatic cancer on two levels: within the human population and within populations of the neoplastic cells themselves. Although the neoplastic cells themselves are not independent organisms, populations of cells do evolve in the face of environmental pressures, and including them in a discussion of evolution leads to new understandings of the growth and dissemination of pancreatic cancer. In this perspective, we start with several straightforward examples to demonstrate how the principles of evolution can be applied to human populations and to the cancers themselves, and we then end with what we hope are provocative insights generated when the pathology and genetics of pancreatic cancer are viewed “in the light of evolution.” The ideas presented are intentionally simple, as our goal is to incite reflection, and for readers to apply the principles of evolution to their own work.

The second passage we include from the *Origins of Species By Means of Natural Selection* is more inspirational; one that we hope will motivate the reader to read on.

There is grandeur in this view of life, with its several powers, having been originally breathed into a few forms or into one; and that, whilst this planet has gone cycling on according to the fixed law of gravity, from so simple a beginning endless forms most beautiful and most wonderful have been, and are being, evolved [5].

## 2 Inherited genetic variants

### 2.1 Highly deleterious germline variants *vs*. variants with small effect

The first example we will give is a very simple one, but it nicely illustrates the power of an evolutionary approach. Pancreatic cancer runs in some families, and a number of the genetic loci associated with an increased risk of pancreatic cancer have been identified [6]. Some of these loci have been discovered using candidate gene or unbiased sequencing approaches such as whole genome and whole exome sequencing, while others have been found through genome-wide association studies (GWAS) (Table 1) [6–14].

Of the established pancreatic cancer susceptibility genes, the penetrance of pancreatic cancer is highest in patients with pathogenic germline variants in *STK11*, the gene that causes the Peutz-Jeghers syndrome (Table 1) [6, 15]. The increased risk of pancreatic cancer in individuals with the Peutz-Jeghers syndrome is up to 135-fold greater than the risk of the general population [16–18]. This translates to a remarkable lifetime risk of developing pancreatic cancer of close to 60%. Fortunately, Peutz-Jeghers syndrome is extremely rare, with an incidence of between 1 in 25,000 and 1 in 300,000 births in the USA [19].

**Fig. 1:**
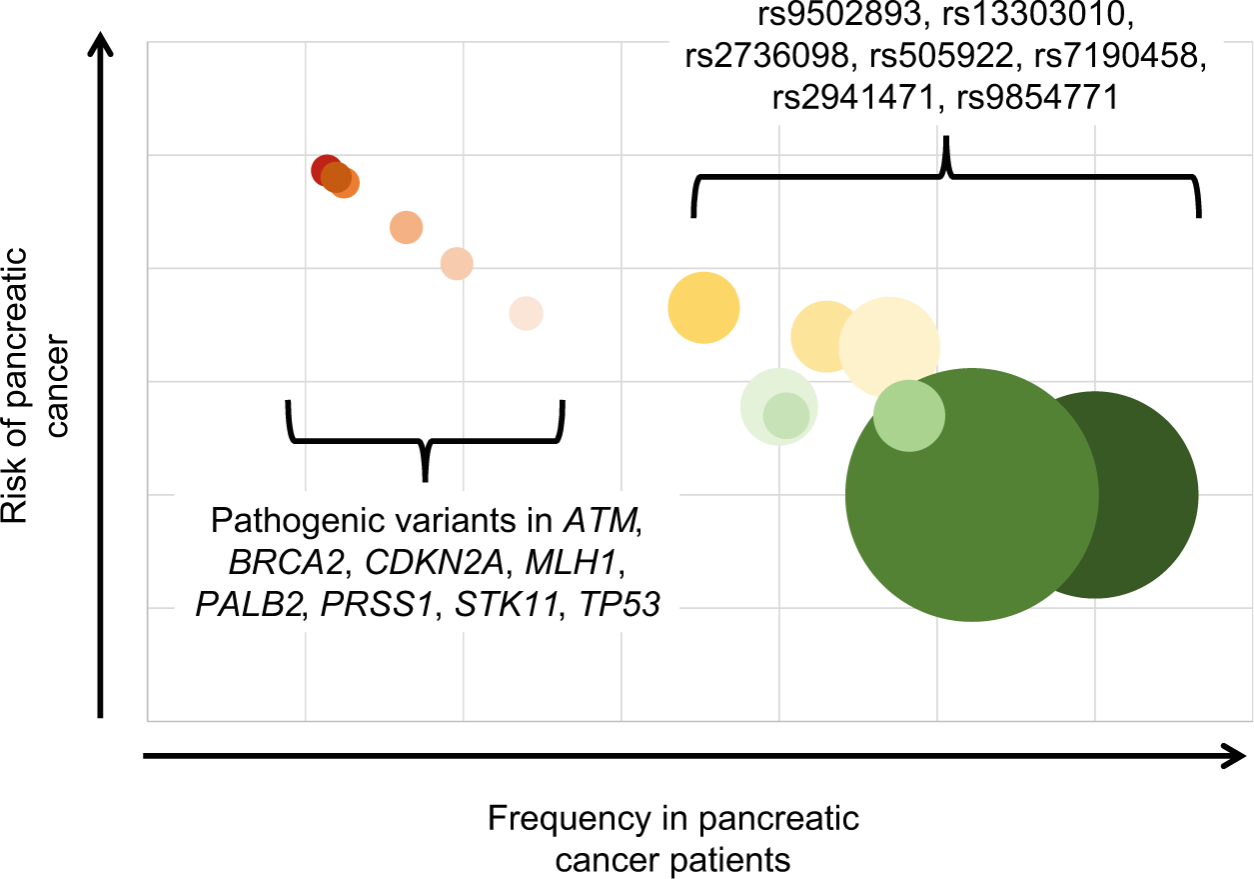
Graphical representation of variants associated with risk of pancreatic cancer. Graph shows pancreatic cancer risk and prevalence of germline variant in patients with pancreatic cancer. Not all variants associated with risk of pancreatic cancer are shown. Size of each circle represents the prevalence of variant in general population. Rare high-risk variants in red. Common low-risk variants in green

By contrast to the rare highly penetrant germline variants in the genes shown in Table 1, common variants of small effect have also been described (Fig. 1). GWAS of large numbers of patients with pancreatic cancer have uncovered a number of loci that increase the risk of pancreatic cancer, but only slightly [10, 12, 20–22]. For example, Amundadottir et al. genotyped 558,542 single nucleotide polymorphisms (SNPs) in 1896 individuals with pancreatic cancer and 1939 controls drawn from 12 prospective cohorts and one hospital-based case-control study [8]. They found that SNP rs505922 on 9q34 was associated with pancreatic cancer (combined *P* = 5.37 × 10^−8^; odds ratio 1.20). This SNP maps to the first intron of the ABO blood group gene. Individuals with blood group O were found to have a lower risk of pancreatic cancer than those with groups A or B. In contrast to the rarity of the Peutz-Jeghers syndrome, 55% of the population is blood group A or B! Fortunately, the odds ratio for developing pancreatic cancer in individuals who have blood group A or B is only 1.2–1.3 [23]. As seen in Fig. 1, when the prevalence of a genetic variant is plotted against the risk it confers of developing pancreatic cancer (penetrance of the variant), a general pattern emerges, in which highly deleterious variants are significantly rarer than less deleterious variants.

Figure 1 makes perfect sense when looked at in the light of evolution. As Darwin noted, “from the strong principle of inheritance, any selected variety will tend to propagate its new and modified form [5].” The converse of this, of course, is that deleterious “varieties” will tend to be eliminated from the population. Individuals with pathogenic germline variants are less likely to survive and, as populations reach equilibrium, these variants become rare. We see this with variants in the *STK11* gene and ABO blood type. Highly deleterious germline changes, which in the case of pancreatic cancer susceptibility genes often increase risk of other lethal diseases with an early age of onset, are eliminated over time (they are rare). While “varieties” of small effect, such as those in the ABO blood group, have more limited impact on survival and are therefore eliminated more gradually (Fig. 1), one would expect that all deleterious alleles (“varieties”), unless they affect only adults long after reproduction, would eventually be eliminated over time. Some alleles that are deleterious in one way may remain in a population if they have some other, matching, benefit, such as the heterozygote advantage of sickle cell anemia in areas where malaria is endemic [24].

### 2.2 Founder effects increase risk in specific populations

We have seen that high-penetrant pathogenic germline variants are typically eliminated from the population; however, in defined populations, certain pathogenic germline variants can be frequent. For example, approximately 1% of individuals of Ashkenazi Jewish decent have a pathogenic germline *BRCA2* variant (c.6174delT) that is associated with a 10-fold increased risk of pancreatic cancer, as well as an increased risk of other cancers including cancers of the breast, ovary, and prostate [25–30]. This is in comparison to the 0.04% of the general European population that are carriers of the same variant [25–30]. Similarly, the prevalence of certain *CDKN2A* variants is higher in specific populations of some countries and regions, indicating founder effects. For example, the pathogenic germline variants p.M53I, p.G101W, p.V126D, and c.225_243del are present in up to 22%, 60%, 90%, and 14% of British, Southern European, Dutch, and North American families with a pathogenic germline *CDKN2A* variant respectively [31].

**Fig. 2:**
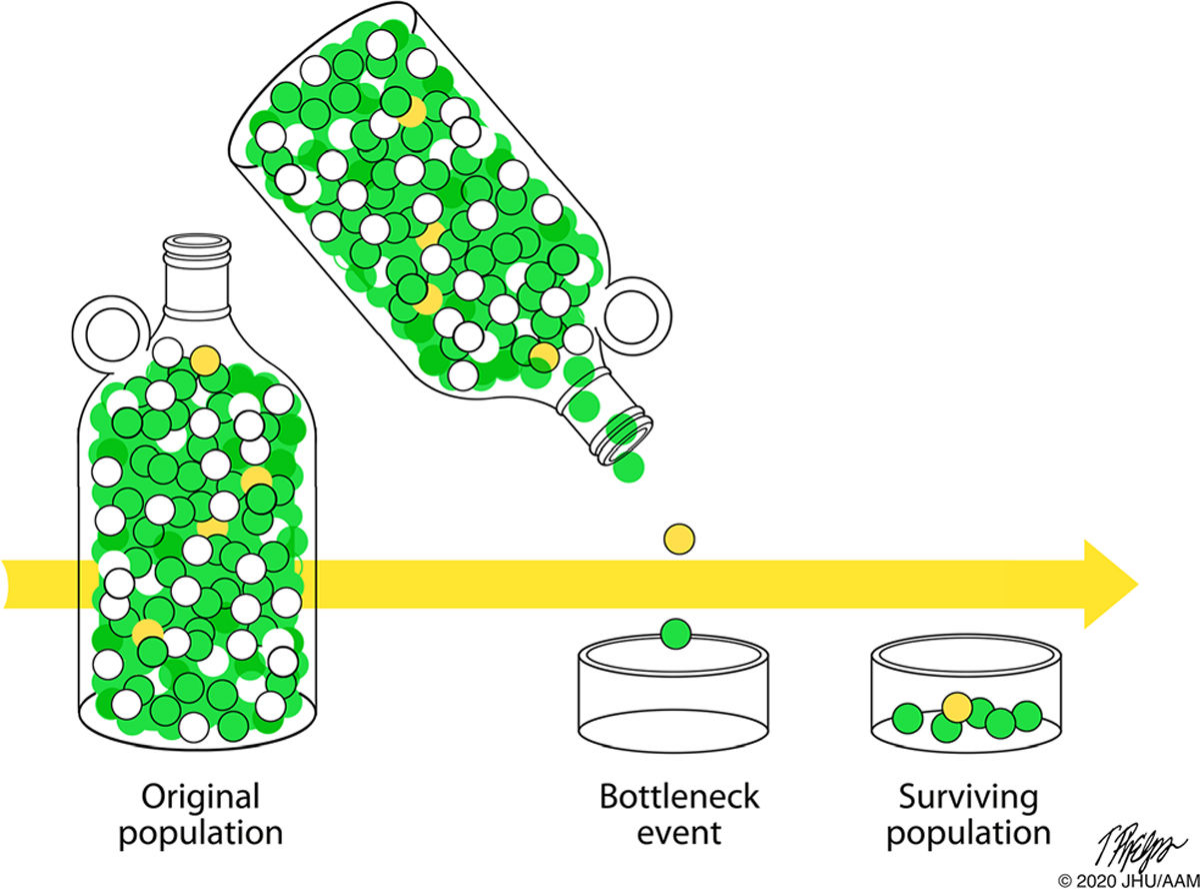
The prevalence of a rare genetic variant (yellow balls) will increase significantly if it happens to pass through a population bottleneck

Why, paradoxically, do some highly penetrant pathogenic germline variants become frequent in a population, even though they are deleterious? Again, the light of evolution provides insight into this conundrum. Consider, for instance, the *BRCA2* variant c.6174delT that is prevalent in individuals of Ashkenazi Jewish decent. Most modern-day Ashkenazi Jews are descended from as few as 350 people who left the Middle East less than 2000 years ago [32–39]. The main population expansion likely occurred after 1000 A.C.E., and this was then followed by additional population bottlenecks in the Middle Ages [32–36, 39]. Ashkenazi Jews who survived these bottlenecks contributed a large percentage of the gene pool present in present-day Ashkenazi Jews. The *BRCA2 c*.6174delT germline variant likely arose in an Ashkenazi Jew 29 generations (~ 750 years) ago [37–39]. It is likely that this initially rare germline genetic variant passed through one of the bottlenecks and, in so doing, remained in a significant percentage of the Ashkenazi Jewish population (Fig. 2) [32–36].

## 3 Somatic mutations in primary cancers

Our cells accumulate large numbers of mutations every day. There are ~ 37.2 trillion human cells in the human body, and most of these cells divide on a regular basis [40–44]. For example, each human produces around 10^11^ (100,000,000,000) new blood cells and billions of other types of cells each day [45]. As cells divide, they need to copy their DNA, and when they do, two to ten mutations are estimated to occur per diploid genome per cell division [46–48]. Multiply this by the billions of cells undergoing division in our bodies each day, and the result is the accumulation of billions of somatic mutations each day!

It should therefore not be surprising that cancers harbor large numbers of somatic mutations [49–51]. Recent whole genome sequencing of large series of pancreatic cancers identified an average mutational burden of one somatic mutation per 2.64 Mb (range 0.65–28.2 per Mb), where the vast majority of the mutations are passenger mutations [52]. This raises several thought-provoking questions that, again, are best answered in the light of evolution.

### 3.1 Why do our cells accumulate these somatic mutations?

**Fig. 3:**
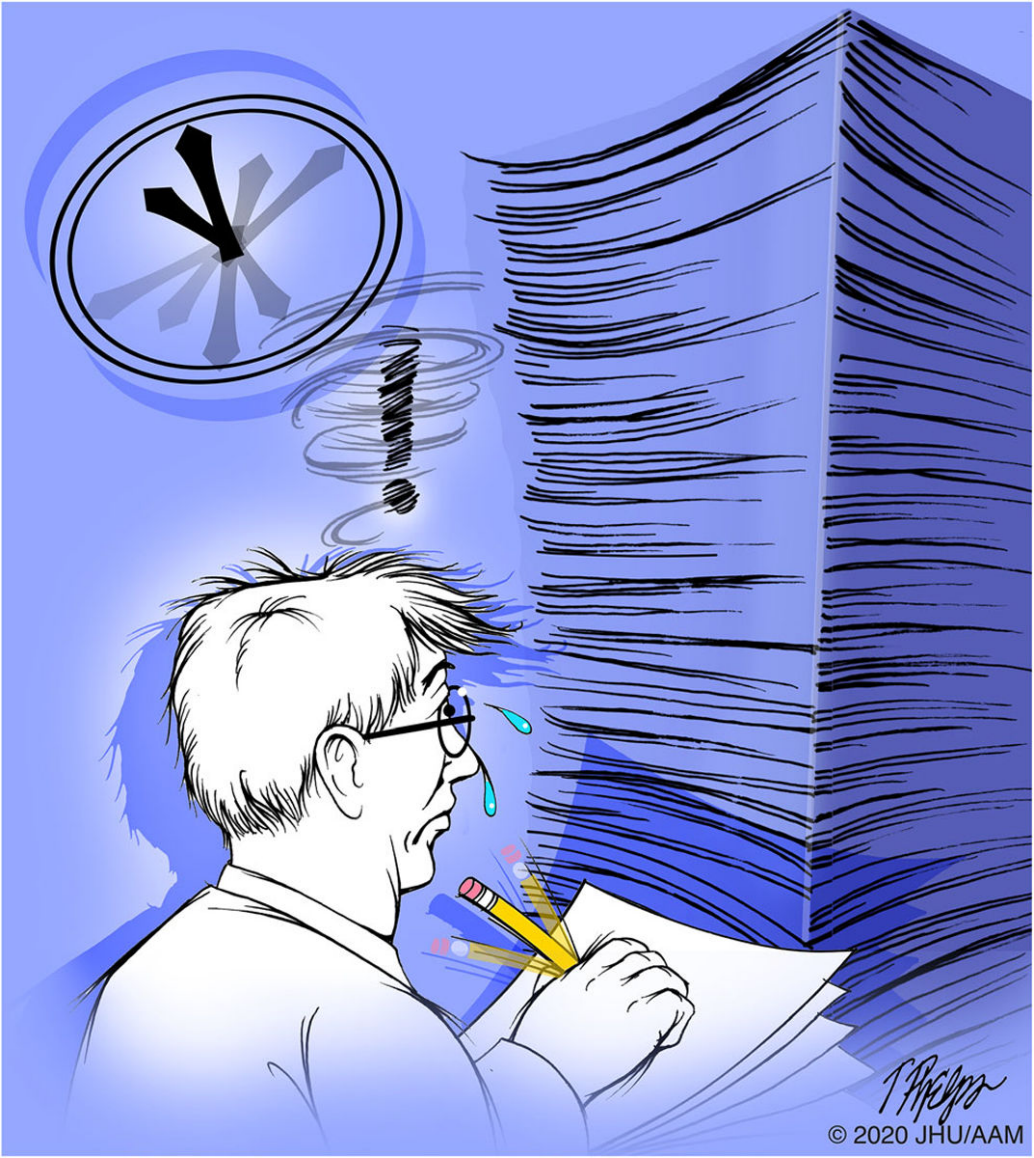
Proof-reading DNA replication is costly to cells in terms of energy and time

Why, with billions of years of evolution to perfect DNA replication, do our cells still make so many errors? Why don’t cells correct somatic mutations as they occur? The answer is simple when viewed in the light of evolution. Imperfect DNA replication allows for evolution to occur. If we copied our germline DNA perfectly, we would never be able to evolve; we would still be slime on a primordial pond! In addition, proof-reading DNA replication is costly to cells in terms of energy and time (Fig. 3) [53, 54]. Gradual improvement in the fidelity of polymerases would also work against the goal of evolving a perfect polymerase: selecting for higher fidelity polymerases decreases the mutation rate, thereby preventing new mutations that would be required in order to increase the fidelity of the polymerases even further [55]. Simply put, mutation rates reflect an evolutionary balance between the costs and benefits of allowing mutations to accumulate [53].

### 3.2 Why doesn’t our immune system eliminate all neoplasms?

Our immune system can be effective in eliminating early neoplasms, so why doesn’t it eliminate all neoplasms [56]? The answer is, in part, because our immune system is also a product of evolution. As our immune system has evolved, it has faced the evolutionary balance between immune surveillance and autoimmunity [57–59]. Our immune system has evolved to recognize certain antigens and not others. Diversity of antibodies and T cell receptors has evolved to neutralize an ever-changing variety of pathogens, and negative selection has evolved to prevent the recognition of self-antigens. The recognition of too many epitopes can lead to autoimmunity, and failure to recognize antigens can lead to infections and to the emergence of a cancer [60]. Individuals who are born with a stronger immune system will be better able to fight infections (and cancer), while those who are born with a weaker immune system will be less likely to develop autoimmune diseases. Those individuals with an immune system, as Darwin wrote, “in any manner profitable to itself,” will have a better chance of surviving. When viewed in the light of evolution, the immune surveillance of cancer is therefore imperfect, because natural selection balances the benefits of immune surveillance with the costs of autoimmunity [57, 61]. Conversely, as cancers develop, the neoplastic cells that successfully evade the immune system will be selected for and we would predict that advanced cancers would be relatively resistant to immunotherapies [56].

## 4 The emergence of resistant clones after targeted therapy

**Fig. 4:**
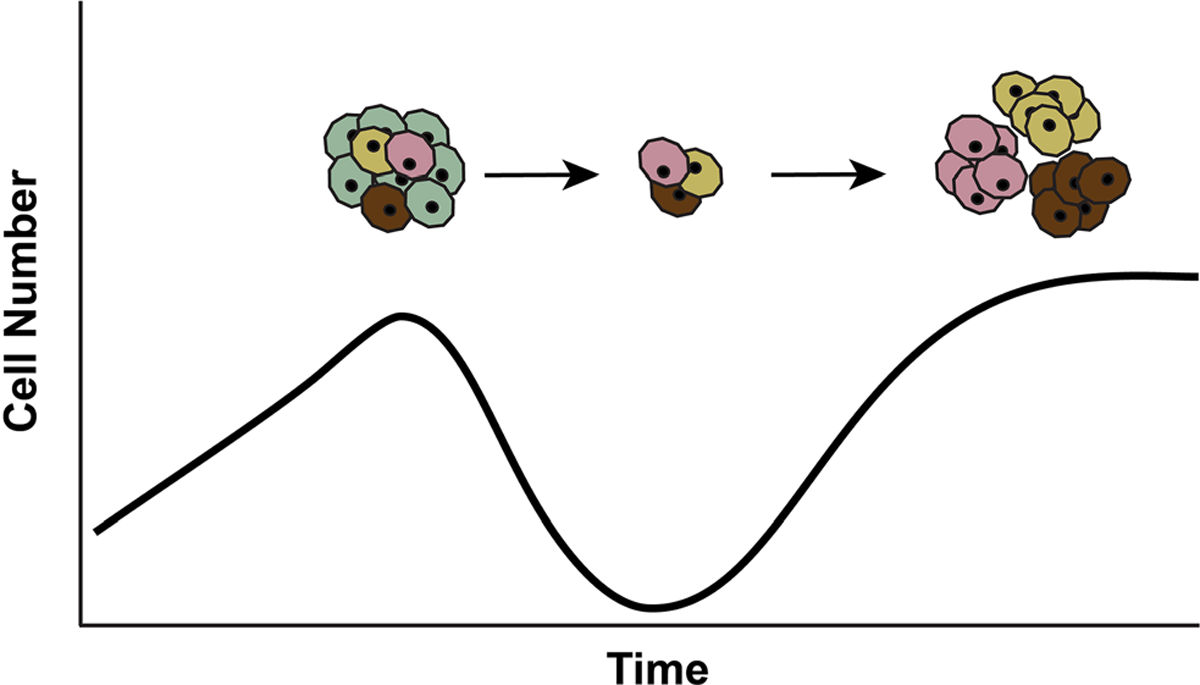
Although therapies may successfully kill most of the neoplastic cells, small populations of pre-existing neoplastic cells with genetic alterations that allow them to survive the selective pressure of the therapy will emerge to form drug-resistant clones

The emergence of resistance has been recognized as a major clinical problem in the treatment of infectious diseases since antibiotics were first developed. The treatment of tuberculosis in the 1950s and of human immunodeficiency virus (HIV) in the 1990s are great examples [62]. The same holds true for the treatment of cancer [3, 63–66]. Resistance often emerges quickly under the selective pressures of targeted therapies, as cells with genetic variants that provide resistance against the therapy will have an increased fitness and will be selected for (Fig. 4). For example, some melanomas are driven by somatic *BRAF* gene mutations, and the introduction of effective BRAF inhibitors offered great hope for the treatment of melanoma. Unfortunately, although great initial responses were seen in human trials, melanomas treated with only a targeted BRAF inhibitor almost always recurred [67]. Remarkably, in some patients with multiple metastases, essentially, every single one of the many metastatic nodules recurred [67].

We can anticipate the same problem with targeted therapies for pancreatic cancer. Some pancreatic cancers with biallelic inactivation of the *BRCA2* gene respond dramatically to poly (ADP-ribose) polymerase (PARP) inhibitors [68–70]. But experience has shown that when cancers with biallelic inactivation of *BRCA2* are treated with an agent that exploits this genetic vulnerability (such as a PARP inhibitor), clones will emerge that harbor additional intragenic *BRCA2* mutations that restore the reading frame and in so doing restore BRCA2 function [69, 71]. These cells with secondary mutations in *BRCA2* will be resistant to therapy and they will grow, leading to the emergence of clinical resistance to that targeted therapy.

The mutations that arise in patients with recurrent and metastatic PDAC after other therapies are only beginning to be characterized [72]. Some of these mutations arise in genes rarely if ever mutated in primary untreated pancreatic cancers. Not surprisingly, these mutations are typically were present in pre-existing clones and provide additional oncogenic advantage or help to overcome environmental pressures such as targeted therapies. Examples of such mutated genes include genes coding for members of the MEK-ERK pathway and for the PI3K-MTOR pathway [72]. Finally, heterogeneity of mutated driver genes can exist within different metastatic clones, indicating that different tumor microenvironments can drive the selection of distinct subclones which may result in different adaptations to targeted therapies in different metastatic sites [72].

This emergence of resistance can be understood when viewed with the light of evolution. Advanced cancers are the products of hundreds to thousands of cell generations and contain a billion to a trillion (10^12^) cells. By chance, one of these billions of cells is likely to acquire a somatic genetic alteration that makes that cell and its descendants resistant to a targeted therapy [73]. These resistant cells will remain a small subpopulation in the cancer until the patient is treated with the targeted therapy (Fig. 4). Targeted therapies effectively kill vulnerable cells, creating powerful pressures that select for clones that have acquired resistance to the therapy. Again, quoting Darwin, “it is not the strongest of the species that survives, nor the most intelligent that survives. It is the one that is most adapted to its present environment [5].” Targeted cancer therapy provides a powerful selective environment for cancer cells which happen to have a mutation that confers resistance to the therapy.

The lesson that evolution teaches us here is the same taught when single agents were first used to treat tuberculosis or HIV [62]. Single agents for these diseases will not succeed, as only a single mutation can quickly lead to the emergence of resistant clones. Single-agent targeted therapies to treat pancreatic cancer will similarly fail. Combination therapies that incorporate agents that target distinct pathways are more likely to succeed as it is unlikely that pre-existing clones exist in a cancer that have mutations that simultaneously confer resistance to multiple different therapies. Taking it one step further, Walther and colleagues have suggested that lessons from the extinction of species can be used to guide novel approaches to drive tumors “to extinction [74].”

## 5 Provocative thoughts

In addition to the more obvious examples given above, the light of evolution can provide provocative insights into the biology of pancreatic cancer.

### 5.1 Traits should not be considered in isolation

**Fig. 5:**
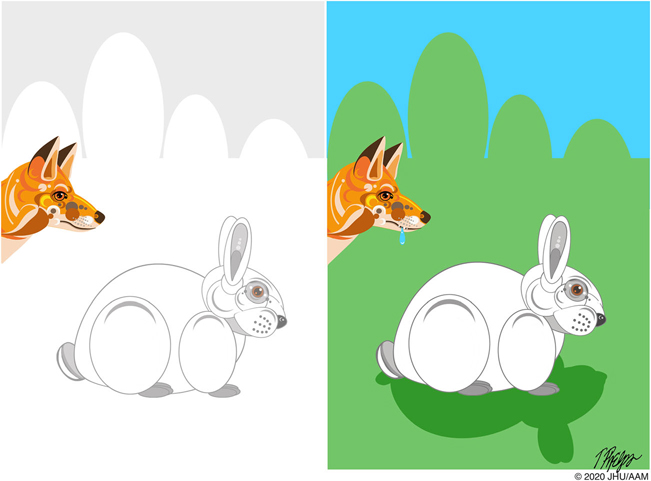
A trait that provides a selective advantage in one environment (white fur in the snow) may not provide a selective advantage in another (white fur on green grass)

Darwin emphasized that a trait that provides a selective advantage in one environment may not provide a selective advantage in another (Fig. 5):

…when a plant or animal is placed in a new country among new competitors…the conditions of its life will greatly be changed in an essential manner. If we wished to increase its average numbers in its new home, we shall have to modify it in a different way to what we should have had to do in its native country [5].

If we apply this reasoning to the progression of pancreatic neoplasia, we can see that genetic alterations that are important in the development of precursor lesions, such as pancreatic intraepithelial neoplasia (PanIN) lesions and intraductal papillary mucinous neoplasms (IPMNs), may no longer be important when that same cell, or its descendants, have metastasized and are present in a completely different environment, such as in the liver [3, 65, 75]. Traits that give a cell a survival advantage in the confines of a duct in the pancreas are unlikely to provide the same survival advantage in the dramatically different environment of the liver. For example, an activating point mutation in *KRAS* may provide a survival advantage to a cell in the pancreatic duct [76–80]. This cell may then grow into a PanIN lesion and subsequently into an invasive carcinoma which then metastasizes to other organs [81]. All of the cells in the metastases will harbor an identical *KRAS* mutation, but, as observed in experimental models, this doesn’t mean that the neoplastic cells in their new environments are still necessarily dependent on mutant *KRAS* for their growth advantage [82, 83]. This possibility has significant implications for targeted therapy, as, for example, the impact of targeting *KRAS* in distant organs may be very different from that of targeting *KRAS* in PanIN lesions [84, 85].

Another striking example of this phenomenon is the distinct prevalence of mutations in specific driver genes at different stages of tumorigenesis. Some driver genes, such as *TP53* and *SMAD4*, are mutated at much higher prevalence in advanced precursor lesions and invasive carcinomas, suggesting that selection for these mutations is limited to late in pancreatic tumorigenesis [86]. In contrast, recent studies have demonstrated mutations in other driver genes at a higher prevalence in early neoplasia. For example, mutations in *RNF43* occur at higher prevalence in noninvasive IPMNs than in invasive carcinomas associated with an IPMN, suggesting that the mutations are selected during precancerous neoplasia but selected against in the subclone that eventually invades [87]. Similarly, hotspot mutations in *KLF4* occur at higher prevalence in low-grade IPMNs than in high-grade IPMNs and are frequently limited to low-grade regions in IPMNs with both grades [88]. These observations suggest that *KLF4* mutations are selected for in low-grade IPMNs but selected against during progression to high-grade dysplasia. However, the mechanisms and evolutionary drivers underpinning this differential selection have yet to be identified.

### 5.2 Phenotypic and genetic drift can be confused for growth advantage

**Fig. 6:**
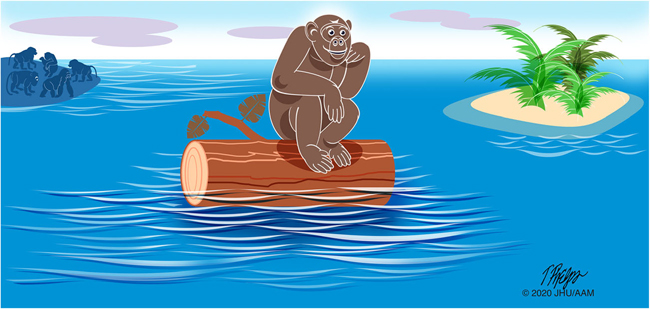
The chance survival of one member of a population can lead to genetic drift. Sampling that population at a later date may give the erroneous impression that the population was selected for because it had a survival advantage

Not all of the phenotypes and genotypes observed in a population provide a selective advantage [89–92]. Genetic drift describes the random fluctuations in gene variants in a population, due to the random sampling when a variant is passed on from one generation to the next. As shown in Fig. 6, genetic drift is most easily detected when individuals are isolated from the main group.

Extending this to pancreatic neoplasia, we can surmise that not all of the genotypes and phenotypes we observe in a tumor have clinical or biological significance [90]. For example, if we are therapeutically targeting a genetic change that is present in one area of a cancer, we need to realize that this genetic change may well be a passenger mutation that occurred late in the development of the invasive cancer [48, 90, 93, 94].

This problem is illustrated in the hunt for genetic alterations that “drive” metastases. A number of investigators have looked for drivers of metastases by comparing the genetic changes in metastases to paired primary lesions from the same patients [95]. This approach assumes that any genetic changes in the metastases and not in the primary lesion must be promoting the metastases. While it is human nature to assume that any differences are causal, Darwinian principles tell us that we must also consider chance spread with genetic drift (Fig. 6). Indeed, although there are differences between metastases and primary lesions, no “metastasis gene” has yet been discovered [80, 95]. This perhaps should not be surprising as D. Shibata and colleagues have shown, using multiregional sequencing of invasive colon cancer, that barriers to invasion are minimal, suggesting that there aren’t “late bottlenecks” in cancer evolution [96].

The same is also true for phenotypes. When pathologists look at histologic sections, many of the morphologic changes observed will not have biological or clinical significance. For example, some pancreatic cancers have a “clear cell” and others a “foamy gland” appearance [97, 98]. While these distinctive appearing cancer types have caught the eye of pathologists, they have no known biological or clinical importance.

Even though many features do not have an adaptive advantage, it is human nature to ascribe significance to each feature we observe.

### 5.3 We do not completely understand gene-environment interactions

The selective pressures placed on a species by its changing environment are difficult to comprehend fully [99]. As Darwin wrote, “we are much too ignorant in regard to the whole economy of any one organic being, to say what slight modifications would be of importance or not… we may sometimes attribute importance to characters which are really of very little importance, and which have originated from quite secondary causes, independently of natural selection [5].”

The complexity of interactions with the environment are highlighted in pancreatic cancer. It was assumed for years that the intense desmoplastic stroma elicited by invasive pancreatic cancers is bad for the patient, that it prevents therapies from reaching the cancer cells, and that if we could only eliminate the stromal environment, we would be able to cure the cancers [100]. Unfortunately, the role of the stroma, the “environment” of pancreatic cancer, has proven enormously complex in pancreatic cancer [100, 101]. Some attempts to reduce the stroma have actually promoted tumor growth and metastases [102]. Moreover, recent work suggests that pancreatic cancer cells that have invaded back into the ductal system are less responsive to chemotherapy than cancer cells in the stroma [103]. We clearly do not fully understand the gene-environment interactions that drive pancreatic cancer.

### 5.4 Most “varieties” never emerge through all of the bottlenecks

**Fig. 7:**
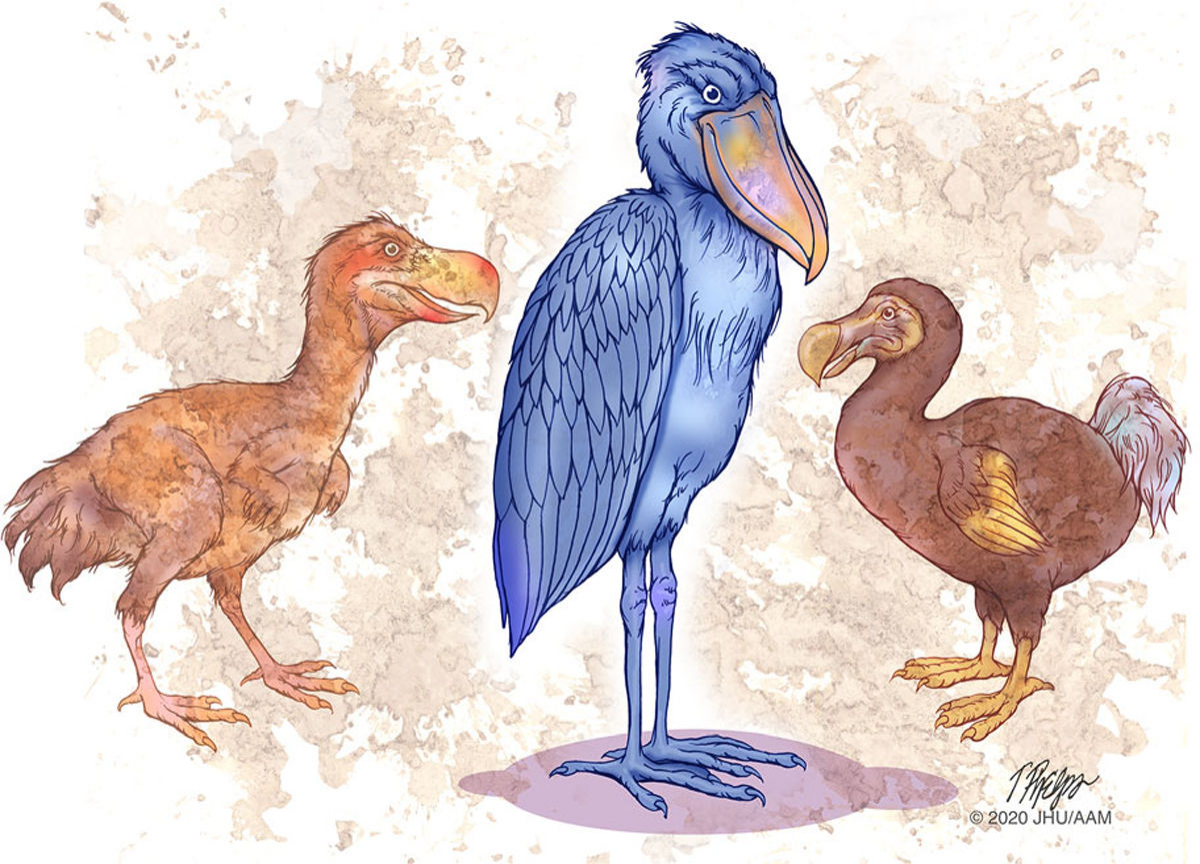
Most “varieties” never emerge through all of the bottlenecks. Pictured are *Titanis* known as the “terror” bird, a shoebill stork, and the dodo. All are extinct except for the shoebill

We can learn from evolution that many new variants are initiated but the promotion of a variant to a separate species is rare (Fig. 7). By analogy, humans develop a large number of precancerous neoplasms, and yet most of these neoplasms never progress through all the bottlenecks to invasive carcinoma. Simply put, as we age, neoplasms are commonly or even ubiquitously initiated but promotion to full malignancy is rare [104–106].

This has profound implications for the early detection of early pancreatic neoplasia as most of the precancers detected will never progress to invasive cancer, and we face a real risk of over treating these clinically harmless precursor lesions [107]. For example, 54% of “normal” adult pancreata have at least one PanIN lesion [108], and 2.6% of patients without pancreatic symptoms have a pancreatic IPMN detectable on computerized tomography scanning [109]. Clearly, the vast majority of individuals with one of these precancerous lesions will never develop invasive pancreatic cancer [110]. Yet, when we detect these lesions, there is a natural desire to remove them, and, as a result, some patients are over treated [111–113].

This variation in the behavior of precancerous lesions is reflected in the significant genetic heterogeneity within and among precursor lesions. Wood and colleagues carefully microdissected invasive cancers that arose in pancreata with a well-defined intraductal papillary mucinous neoplasm (IPMN) and found that IPMNs are genetically heterogeneous and that the invasive cancers sometimes were genetically unrelated to the IPMNs [76, 87, 114, 115]. Thus, it is clear that some pancreata harbor many heterogeneous noninvasive neoplastic clones and that only a small fraction of these clones progress to invasion [116–118]. Within these precancerous neoplasms, some patterns in the genetic heterogeneity can be discerned. For example, heterogeneity with respect to mutations in the initiating driver gene *KRAS* and *GNAS* occurs in IPMNs with low-grade dysplasia, indicating polyclonal origin [114]. In contrast, high-grade IPMNs often contain monoclonal populations of neoplastic cells, but subclones within these populations contain different mutations in the same tumor suppressor gene. In particular, this pattern has been described in mutations in the tumor suppressor gene *RNF43*. A single IPMN can acquire multiple *RNF43* mutations, each limited to a distinct subclone. This pattern suggests parallel evolution with respect to these mutations, i.e., the independent evolution of similar features due to common function, in this case in distinct neoplastic subclones [87, 114, 119]. Taken together, these findings suggest complex selective pressures that vary throughout the different stages of preinvasive pancreatic neoplasia. This genetic heterogeneity is counterintuitive to a simpler model of a series of sequential mutations in oncogenes and tumor suppressor genes that invariably progresses to an invasive cancer. Still, emerging data suggest that premalignant tumorigenesis occurs *via* waves of clonal diversity, followed by bottlenecking, as strongly selected driver mutations arise during the carcinogenic process [87, 114, 119]. Finally, invasion can also be considered as a founder effect: a cell escapes the highly competitive and heterogeneous environment of the precursor lesion, to find new resources and space to grow. The lack of clonal heterogeneity in invasive cancer and metastases might indicate that this is the last bottleneck.

Although most invasive pancreatic cancers are extremely aggressive, we also cannot completely rule out the unlikely possibility that some minimally invasive pancreatic cancers will not metastasize during the life of the patient. This is nicely illustrated with thyroid cancer, where increased screening has led to the detection and removal of many invasive cancers, but has not reduced the mortality from the disease [120, 121]. It appears that many of the thyroid cancers detected on screening would never have harmed the patient. In the case of the pancreas, the jury is still out as to whether the detection of early, stage I, cancers arising in association with an IPMN saves lives [122].

## 6 Conclusions

It has been more than 185 years since Charles Darwin first set sail on the Beagle, and over 160 years since his masterpiece the *Origins of Species By Means of Natural Selection* was first published in 1859. Darwin’s theory of evolution by means of natural selection is arguably one of the most important ideas ever put forth in the life sciences. The principles laid out by Darwin continue to provide insight into all aspects of biology. Here, in some small way, we hope we have convinced you that “thinking evolutionarily” can provide insight into modern pancreatic genetics and pancreatic pathology.

Since we began with passages from Darwin, we thought we should end with Darwin’s words.

“The distribution of tenants of this archipelago would not be nearly so wonderful, if for instance, one island has a mocking-thrush and a second island some other quite distinct species... But it is the circumstance that several of the islands possess their own species of tortoise, mocking-thrush, finches, and numerous plants, these species having the same general habits, occupying analogous situations, and obviously filling the same place in the natural economy of this archipelago, that strikes me with wonder” (journal written on the Beagle 1831–1835 [5]).

These words prompt us, as pathologists and pancreatic cancer researchers, to more carefully study the complex varieties of pancreatic neoplasms. It is in doing so that we may gain unique insight into this terrible disease.

## Figures and Tables

**Table 1:** Germline variants and the risk of pancreatic cancer

Gene	% of patients with pancreatic cancer	Increased Risk	Age 50^+^	Age 70
SEER USA population	–	1	0.05%	0.5%
Hereditary non-polyposis colorectal cancer (Lynch syndrome) genes	< 1	7–8	0.4%	4%
*ATM*	1–4	6	0.3%	3%
*BRCA2*	2–7	2–6	Up to 0.3%	Up to 3%
*BRCA1*	Up to 1	1–2.5	Up to 0.15%	
*PALB2*	< 1	2.4	0.12%	1.2%
*p16/CDKN2A*	1–3	12–46	Up to 2.3%	Up to 23%
*PRSS1*	< 1	50–60	3%	30%
*STK11*	< 1	75–135	6.75%	Up to 67.5%
*TP53*	< 1	6–7	0.35%	Up to 3.5%

+ Using the high end of the range of increased risk

## References

[1] Dobzhansky T (1937). Genetics and the origin of species. New York: Columbia University Press.

[2] Ayala FJ, & Fitch WM (1997). Genetics and the origin of species: an introduction. Proceedings of the National Academy of Sciences of the United States of America, 94(15), 7691–7697. 9223250 10.1073/pnas.94.15.7691 PMC33678

[3] Greaves M (2018). Nothing in cancer makes sense except. BMC Biology, 16(1), 22. 29466995 10.1186/s12915-018-0493-8 PMC5822549

[4] Nowell PC (1976). The clonal evolution of tumor cell populations. Science, 194(4260), 23–28. 959840 10.1126/science.959840

[5] Darwin C (1859). On the origin of species by means of natural selection, on the preservation of favoured races in the struggle for life. London: John Murray. PMC5184128 30164232

[6] Thompson ED, Roberts NJ, Wood LD, Eshleman JR, Goggins MG, Kern SE, Klein AP, & Hruban RH (2020). The genetics of ductal adenocarcinoma of the pancreas in the year 2020: dramatic progress, but far to go. Modern Pathology, 33, 2544–2563. 32704031 10.1038/s41379-020-0629-6 PMC8375585

[7] Roberts NJ, Norris AL, Petersen GM, Bondy ML, Brand R, Gallinger S, Kurtz RC, Olson SH, Rustgi AK, Schwartz AG, Stoffel E, Syngal S, Zogopoulos G, Ali SZ, Axilbund J, Chaffee KG, Chen YC, Cote ML, Childs EJ, Douville C, Goes FS, Herman JM, Iacobuzio-Donahue C, Kramer M, Makohon-Moore A, McCombie RW, McMahon KW, Niknafs N, Parla J, Pirooznia M, Potash JB, Rhim AD, Smith AL, Wang Y, Wolfgang CL, Wood LD, Zandi PP, Goggins M, Karchin R, Eshleman JR, Papadopoulos N, Kinzler KW, Vogelstein B, Hruban RH, & Klein AP (2016). Whole genome sequencing defines the genetic heterogeneity of familial pancreatic cancer. Cancer Discovery, 6(2), 166–175. 26658419 10.1158/2159-8290.CD-15-0402 PMC4744563

[8] Amundadottir L, Kraft P, Stolzenberg-Solomon RZ, Fuchs CS, Petersen GM, Arslan AA, Bueno-de-Mesquita HB, Gross M, Helzlsouer K, Jacobs EJ, LaCroix A, Zheng W, Albanes D, Bamlet W, Berg CD, Berrino F, Bingham S, Buring JE, Bracci PM, Canzian F, Clavel-Chapelon F, Clipp S, Cotterchio M, de Andrade M, Duell EJ, Fox JW Jr, Gallinger S, Gaziano JM, Giovannucci EL, Goggins M, González CA, Hallmans G, Hankinson SE, Hassan M, Holly EA, Hunter DJ, Hutchinson A, Jackson R, Jacobs KB, Jenab M, Kaaks R, Klein AP, Kooperberg C, Kurtz RC, Li D, Lynch SM, Mandelson M, McWilliams RR, Mendelsohn JB, Michaud DS, Olson SH, Overvad K, Patel AV, Peeters PHM, Rajkovic A, Riboli E, Risch HA, Shu XO, Thomas G, Tobias GS, Trichopoulos D, van den Eeden SK, Virtamo J, Wactawski-Wende J, Wolpin BM, Yu H, Yu K, Zeleniuch-Jacquotte A, Chanock SJ, Hartge P, & Hoover RN (2009). Genome-wide association study identifies variants in the ABO locus associated with susceptibility to pancreatic cancer. Nature Genetics, 41(9), 986–990. 19648918 10.1038/ng.429 PMC2839871

[9] Chen F, Childs EJ, Mocci E, Bracci P, Gallinger S, Li D, Neale RE, Olson SH, Scelo G, Bamlet WR, Blackford AL, Borges M, Brennan P, Chaffee KG, Duggal P, Hassan MJ, Holly EA, Hung RJ, Goggins MG, Kurtz RC, Oberg AL, Orlow I, Yu H, Petersen GM, Risch HA, & Klein AP (2019). Analysis of heritability and genetic architecture of pancreatic cancer: a PanC4 study. Cancer Epidemiology, Biomarkers & Prevention, 28(7), 1238–1245. 10.1158/1055-9965.EPI-18-1235 PMC6606380 31015203

[10] Childs EJ, Mocci E, Campa D, Bracci PM, Gallinger S, Goggins M, Li D, Neale RE, Olson SH, Scelo G, Amundadottir LT, Bamlet WR, Bijlsma MF, Blackford A, Borges M, Brennan P, Brenner H, Bueno-de-Mesquita HB, Canzian F, Capurso G, Cavestro GM, Chaffee KG, Chanock SJ, Cleary SP, Cotterchio M, Foretova L, Fuchs C, Funel N, Gazouli M, Hassan M, Herman JM, Holcatova I, Holly EA, Hoover RN, Hung RJ, Janout V, Key TJ, Kupcinskas J, Kurtz RC, Landi S, Lu L, Malecka-Panas E, Mambrini A, Mohelnikova-Duchonova B, Neoptolemos JP, Oberg AL, Orlow I, Pasquali C, Pezzilli R, Rizzato C, Saldia A, Scarpa A, Stolzenberg-Solomon RZ, Strobel O, Tavano F, Vashist YK, Vodicka P, Wolpin BM, Yu H, Petersen GM, Risch HA, & Klein AP (2015). Common variation at 2p13.3, 3q29, 7p13 and 17q25.1 associated with susceptibility to pancreatic cancer. Nature Genetics, 47(8), 911–916. 26098869 10.1038/ng.3341 PMC4520746

[11] Jiao Y, Lumpkins K, Terhune J, Hruban RH, Klein A, Kinzler KW, Papadopoulos N, Vogelstein B, & Strauch E (2015). Intraductal papillary mucinous neoplasm in a neonate with congenital hyperinsulinism and a de novo germline SKIL gene mutation. Pancreatology, 15(2), 194–196. 25464936 10.1016/j.pan.2014.10.009 PMC4387019

[12] Klein AP, Wolpin BM, Risch HA, Stolzenberg-Solomon RZ, Mocci E, Zhang M, Canzian F, Childs EJ, Hoskins JW, Jermusyk A, Zhong J, Chen F, Albanes D, Andreotti G, Arslan AA, Babic A, Bamlet WR, Beane-Freeman L, Berndt SI, Blackford A, Borges M, Borgida A, Bracci PM, Brais L, Brennan P, Brenner H, Bueno-de-Mesquita B, Buring J, Campa D, Capurso G, Cavestro GM, Chaffee KG, Chung CC, Cleary S, Cotterchio M, Dijk F, Duell EJ, Foretova L, Fuchs C, Funel N, Gallinger S, Gaziano M, J. M., Gazouli M, Giles GG, Giovannucci E, Goggins M, Goodman GE, Goodman PJ, Hackert T, Haiman C, Hartge P, Hasan M, Hegyi P, Helzlsouer KJ, Herman J, Holcatova I, Holly EA, Hoover R, Hung RJ, Jacobs EJ, Jamroziak K, Janout V, Kaaks R, Khaw KT, Klein EA, Kogevinas M, Kooperberg C, Kulke MH, Kupcinskas J, Kurtz RJ, Laheru D, Landi S, Lawlor RT, Lee IM, LeMarchand L, Lu L, Malats N, Mambrini A, Mannisto S, Milne RL, Mohelníková-Duchoňová B, Neale RE, Neoptolemos JP, Oberg AL, Olson SH, Orlow I, Pasquali C, Patel AV, Peters U, Pezzilli R, Porta M, Real FX, Rothman N, Scelo G, Sesso HD, Severi G, Shu XO, Silverman D, Smith JP, Soucek P, Sund M, Talar-Wojnarowska R, Tavano F, Thornquist MD, Tobias GS, van den Eeden SK, Vashist Y, Visvanathan K, Vodicka P, Wactawski-Wende J, Wang Z, Wentzensen N, White E, Yu H, Yu K, Zeleniuch-Jacquotte A, Zheng W, Kraft P, Li D, Chanock S, Obazee O, Petersen GM, & Amundadottir LT (2018). Genome-wide meta-analysis identifies five new susceptibility loci for pancreatic cancer. Nature Communications, 9(1), 556. 10.1038/s41467-018-02942-5 PMC5805680 29422604

[13] Roberts NJ, Jiao Y, Yu J, Kopelovich L, Petersen GM, Bondy ML, Gallinger S, Schwartz AG, Syngal S, Cote ML, Axilbund J, Schulick R, Ali SZ, Eshleman JR, Velculescu VE, Goggins M, Vogelstein B, Papadopoulos N, Hruban RH, Kinzler KW, & Klein AP (2012). ATM mutations in patients with hereditary pancreatic cancer. Cancer Discovery, 2(1), 41–46. 22585167 10.1158/2159-8290.CD-11-0194 PMC3676748

[14] Zhong J, Jermusyk A, Wu L, Hoskins JW, Collins I, Mocci E, (2020). A transcriptome-wide association study (TWAS) identifies novel candidate susceptibility genes for pancreatic cancer. Journal of the National Cancer Institute. 10.1093/jnci/djz246 PMC7566474 31917448

[15] Keller JJ, Offerhaus GJ, Giardiello FM, & Menko FH (2001). Jan Peutz, Harold Jeghers and a remarkable combination of polyposis and pigmentation of the skin and mucous membranes. Familial Cancer, 1(3–4), 181–185. 14574176 10.1023/a:1021149327174

[16] Korsse SE, Harinck F, van Lier MG, Biermann K, Offerhaus GJ, Krak N, (2013). Pancreatic cancer risk in Peutz-Jeghers syndrome patients: a large cohort study and implications for surveillance. Journal of Medical Genetics, 50(1), 59–64. 23240097 10.1136/jmedgenet-2012-101277

[17] Resta N, Pierannunzio D, Lenato GM, Stella A, Capocaccia R, Bagnulo R, Lastella P, Susca FC, Bozzao C, Loconte DC, Sabbà C, Urso E, Sala P, Fornasarig M, Grammatico P, Piepoli A, Host C, Turchetti D, Viel A, Memo L, Giunti L, Stigliano V, Varesco L, Bertario L, Genuardi M, Lucci Cordisco E, Tibiletti MG, di Gregorio C, Andriulli A, Ponz de Leon M, & AIFEG. (2013). Cancer risk associated with STK11/LKB1 germline mutations in Peutz-Jeghers syndrome patients: results of an Italian multicenter study. Digestive and Liver Disease, 45(7), 606–611. 23415580 10.1016/j.dld.2012.12.018

[18] Giardiello FM, Welsh SB, Hamilton SR, Offerhaus GJ, Gittelsohn AM, Booker SV, (1987). Increased risk of cancer in the Peutz-Jeghers syndrome. The New England Journal of Medicine, 316(24), 1511–1514. 3587280 10.1056/NEJM198706113162404

[19] Beggs AD, Latchford AR, Vasen HF, Moslein G, Alonso A, Aretz S, (2010). Peutz-Jeghers syndrome: a systematic review and recommendations for management. Gut, 59(7), 975–986. 20581245 10.1136/gut.2009.198499

[20] Low SK, Kuchiba A, Zembutsu H, Saito A, Takahashi A, Kubo M, Daigo Y, Kamatani N, Chiku S, Totsuka H, Ohnami S, Hirose H, Shimada K, Okusaka T, Yoshida T, Nakamura Y, & Sakamoto H (2010). Genome-wide association study of pancreatic cancer in Japanese population. PLoS One, 5(7), e11824. 20686608 10.1371/journal.pone.0011824 PMC2912284

[21] Wu C, Miao X, Huang L, Che X, Jiang G, Yu D, Yang X, Cao G, Hu Z, Zhou Y, Zuo C, Wang C, Zhang X, Zhou Y, Yu X, Dai W, Li Z, Shen H, Liu L, Chen Y, Zhang S, Wang X, Zhai K, Chang J, Liu Y, Sun M, Cao W, Gao J, Ma Y, Zheng X, Cheung ST, Jia Y, Xu J, Tan W, Zhao P, Wu T, Wang C, & Lin D (2011). Genome-wide association study identifies five loci associated with susceptibility to pancreatic cancer in Chinese populations. Nature Genetics, 44(1), 62–66. 22158540 10.1038/ng.1020

[22] Zhang M, Wang Z, Obazee O, Jia J, Childs EJ, Hoskins J, Figlioli G, Mocci E, Collins I, Chung CC, Hautman C, Arslan AA, Beane-Freeman L, Bracci PM, Buring J, Duell EJ, Gallinger S, Giles GG, Goodman GE, Goodman PJ, Kamineni A, Kolonel LN, Kulke MH, Malats N, Olson SH, Sesso HD, Visvanathan K, White E, Zheng W, Abnet CC, Albanes D, Andreotti G, Brais L, Bueno-de-Mesquita HB, Basso D, Berndt SI, Boutron-Ruault MC, Bijlsma MF, Brenner H, Burdette L, Campa D, Caporaso NE, Capurso G, Cavestro GM, Cotterchio M, Costello E, Elena J, Boggi U, Gaziano JM, Gazouli M, Giovannucci EL, Goggins M, Gross M, Haiman CA, Hassan M, Helzlsouer KJ, Hu N, Hunter DJ, Iskierka-Jazdzewska E, Jenab M, Kaaks R, Key TJ, Khaw KT, Klein EA, Kogevinas M, Krogh V, Kupcinskas J, Kurtz RC, Landi MT, Landi S, le Marchand L, Mambrini A, Mannisto S, Milne RL, Neale RE, Oberg AL, Panico S, Patel AV, Peeters PHM, Peters U, Pezzilli R, Porta M, Purdue M, Quiros JR, Riboli E, Rothman N, Scarpa A, Scelo G, Shu XO, Silverman DT, Soucek P, Strobel O, Sund M, Małecka-Panas E, Taylor PR, Tavano F, Travis RC, Thornquist M, Tjønneland A, Tobias GS, Trichopoulos D, Vashist Y, Vodicka P, Wactawski-Wende J, Wentzensen N, Yu H, Yu K, Zeleniuch-Jacquotte A, Kooperberg C, Risch HA, Jacobs EJ, Li D, Fuchs C, Hoover R, Hartge P, Chanock SJ, Petersen GM, Stolzenberg-Solomon RS, Wolpin BM, Kraft P, Klein AP, Canzian F, & Amundadottir LT (2016). Three new pancreatic cancer susceptibility signals identified on chromosomes 1q32.1, 5p15.33 and 8q24.21. Oncotarget, 7(41), 66328–66343. 27579533 10.18632/oncotarget.11041 PMC5340084

[23] Wolpin BM, Chan AT, Hartge P, Chanock SJ, Kraft P, Hunter DJ, Giovannucci EL, & Fuchs CS (2009). ABO blood group and the risk of pancreatic cancer. Journal of the National Cancer Institute, 101(6), 424–431. 19276450 10.1093/jnci/djp020 PMC2657095

[24] Mangla A, Ehsan M, & Maruvada S (2020). Sickle Cell Anemia. In StatPearls. Treasure Island.

[25] Stadler ZK, Salo-Mullen E, Patil SM, Pietanza MC, Vijai J, Saloustros E, Hansen NAL, Kauff ND, Kurtz RC, Kelsen DP, Offit K, & Robson ME (2012). Prevalence of BRCA1 and BRCA2 mutations in Ashkenazi Jewish families with breast and pancreatic cancer. Cancer, 118(2), 493–499. 21598239 10.1002/cncr.26191

[26] Metcalfe KA, Poll A, Royer R, Llacuachaqui M, Tulman A, Sun P, & Narod SA (2010). Screening for founder mutations in BRCA1 and BRCA2 in unselected Jewish women. Journal of Clinical Oncology, 28(3), 387–391. 20008623 10.1200/JCO.2009.25.0712

[27] Ozcelik H, Schmocker B, Di Nicola N, Shi XH, Langer B, Moore M, (1997). Germline BRCA2 6174delT mutations in Ashkenazi Jewish pancreatic cancer patients. Nature Genetics, 16(1), 17–18. 9140390 10.1038/ng0597-17

[28] Zhen DB, Rabe KG, Gallinger S, Syngal S, Schwartz AG, Goggins MG, Hruban RH, Cote ML, McWilliams RR, Roberts NJ, Cannon-Albright LA, Li D, Moyes K, Wenstrup RJ, Hartman AR, Seminara D, Klein AP, & Petersen GM (2015). BRCA1, BRCA2, PALB2, and CDKN2A mutations in familial pancreatic cancer: a PACGENE study. Genetics in Medicine, 17(7), 569–577. 25356972 10.1038/gim.2014.153 PMC4439391

[29] Murphy KM, Brune KA, Griffin C, Sollenberger JE, Petersen GM, Bansal R, Hruban RH, & Kern SE (2002). Evaluation of candidate genes MAP2K4, MADH4, ACVR1B, and BRCA2 in familial pancreatic cancer: deleterious BRCA2 mutations in 17%. Cancer Research, 62(13), 3789–3793. 12097290

[30] Holter S, Borgida A, Dodd A, Grant R, Semotiuk K, Hedley D, Dhani N, Narod S, Akbari M, Moore M, & Gallinger S (2015). Germline BRCA mutations in a large clinic-based cohort of patients with pancreatic adenocarcinoma. Journal of Clinical Oncology, 33(28), 3124–3129. 25940717 10.1200/JCO.2014.59.7401

[31] Goldstein AM, Chan M, Harland M, Gillanders EM, Hayward NK, Avril MF, Azizi E, Bianchi-Scarra G, Bishop DT, Bressac-de Paillerets B, Bruno W, Calista D, Cannon Albright LA, Demenais F, Elder DE, Ghiorzo P, Gruis NA, Hansson J, Hogg D, Holland EA, Kanetsky PA, Kefford RF, Landi MT, Lang J, Leachman SA, MacKie RM, Magnusson V, Mann GJ, Niendorf K, Newton Bishop J, Palmer JM, Puig S, Puig-Butille JA, de Snoo FA, Stark M, Tsao H, Tucker MA, Whitaker L, Yakobson E, & The Lund Melanoma Study Group. (2006). High-risk melanoma susceptibility genes and pancreatic cancer, neural system tumors, and uveal melanoma across GenoMEL. Cancer Research, 66(20), 9818–9828. 17047042 10.1158/0008-5472.CAN-06-0494

[32] Behar DM, Metspalu E, Kivisild T, Achilli A, Hadid Y, Tzur S, Pereira L, Amorim A, Quintana-Murci L, Majamaa K, Herrnstadt C, Howell N, Balanovsky O, Kutuev I, Pshenichnov A, Gurwitz D, Bonne-Tamir B, Torroni A, Villems R, & Skorecki K (2006). The matrilineal ancestry of ashkenazi jewry: portrait of a recent founder event. American Journal of Human Genetics, 78(3), 487–497. 16404693 10.1086/500307 PMC1380291

[33] Behar DM, Hammer MF, Garrigan D, Villems R, Bonne-Tamir B, Richards M, Gurwitz D, Rosengarten D, Kaplan M, Pergola SD, Quintana-Murci L, & Skorecki K (2004). MtDNA evidence for a genetic bottleneck in the early history of the Ashkenazi Jewish population. European Journal of Human Genetics, 12(5), 355–364. 14722586 10.1038/sj.ejhg.5201156

[34] Carmi S, Hui KY, Kochav E, Liu X, Xue J, Grady F, Guha S, Upadhyay K, Ben-Avraham D, Mukherjee S, Bowen BM, Thomas T, Vijai J, Cruts M, Froyen G, Lambrechts D, Plaisance S, van Broeckhoven C, van Damme P, van Marck H, Barzilai N, Darvasi A, Offit K, Bressman S, Ozelius LJ, Peter I, Cho JH, Ostrer H, Atzmon G, Clark LN, Lencz T, & Pe’er I (2014). Sequencing an Ashkenazi reference panel supports population-targeted personal genomics and illuminates Jewish and European origins. Nature Communications, 5, 4835. 10.1038/ncomms5835 PMC4164776 25203624

[35] Rootsi S, Behar DM, Jarve M, Lin AA, Myres NM, Passarelli B, (2013). Phylogenetic applications of whole Y-chromosome sequences and the Near Eastern origin of Ashkenazi Levites. Nature Communications, 4, 2928. 10.1038/ncomms3928 PMC3905698 24346185

[36] Bray SM, Mulle JG, Dodd AF, Pulver AE, Wooding S, & Warren ST (2010). Signatures of founder effects, admixture, and selection in the Ashkenazi Jewish population. Proceedings of the National Academy of Sciences of the United States of America, 107(37), 16222–16227. 20798349 10.1073/pnas.1004381107 PMC2941333

[37] Neuhausen SL, Godwin AK, Gershoni-Baruch R, Schubert E, Garber J, Stoppa-Lyonnet D, Olah E, Csokay B, Serova O, Lalloo F, Osorio A, Stratton M, Offit K, Boyd J, Caligo MA, Scott RJ, Schofield A, Teugels E, Schwab M, Cannon-Albright L, Bishop T, Easton D, Benitez J, King MC, Ponder BAJ, Weber B, Devilee P, Borg Å, Narod SA, & Goldgar D (1998). Haplotype and phenotype analysis of nine recurrent BRCA2 mutations in 111 families: results of an international study. American Journal of Human Genetics, 62(6), 1381–1388. 9585613 10.1086/301885 PMC1377164

[38] Mozersky J, & Gibbon S (2014). Mapping Jewish identities: migratory histories and the transnational re-framing of ‘Ashkenazi BRCA mutations’ in the UK and Brazil. In Gibbon S, Joseph G, Mozersky J, Nieden AZ, & Palfner S (Eds.), Breast cancer gene research and medical practices: transnational perspectives in the time of BRCA. Oxon: Wellcome Trust-Funded Monographs and Book Chapters.

[39] Im KM, Kirchhoff T, Wang X, Green T, Chow CY, Vijai J, (2011). Haplotype structure in Ashkenazi Jewish BRCA1 and BRCA2 mutation carriers. Human Genetics, 130(5), 685–699. 21597964 10.1007/s00439-011-1003-z PMC3196382

[40] Bianconi E, Piovesan A, Facchin F, Beraudi A, Casadei R, Frabetti F, Vitale L, Pelleri MC, Tassani S, Piva F, Perez-Amodio S, Strippoli P, & Canaider S (2013). An estimation of the number of cells in the human body. Annals of Human Biology, 40(6), 463–471. 23829164 10.3109/03014460.2013.807878

[41] Blokzijl F, de Ligt J, Jager M, Sasselli V, Roerink S, Sasaki N, Huch M, Boymans S, Kuijk E, Prins P, Nijman IJ, Martincorena I, Mokry M, Wiegerinck CL, Middendorp S, Sato T, Schwank G, Nieuwenhuis EES, Verstegen MMA, van der Laan LJW, de Jonge J, IJzermans JNM, Vries RG, van de Wetering M, Stratton MR, Clevers H, Cuppen E, & van Boxtel R (2016). Tissue-specific mutation accumulation in human adult stem cells during life. Nature, 538(7624), 260–264. 27698416 10.1038/nature19768 PMC5536223

[42] Lahouel K, Younes L, Danilova L, Giardiello FM, Hruban RH, Groopman J, Kinzler KW, Vogelstein B, Geman D, & Tomasetti C (2020). Revisiting the tumorigenesis timeline with a data-driven generative model. Proceedings of the National Academy of Sciences of the United States of America, 117(2), 857–864. 31882448 10.1073/pnas.1914589117 PMC6969520

[43] Araten DJ, Golde DW, Zhang RH, Thaler HT, Gargiulo L, Notaro R, & Luzzatto L (2005). A quantitative measurement of the human somatic mutation rate. Cancer Research, 65(18), 8111–8117. 16166284 10.1158/0008-5472.CAN-04-1198

[44] Drake JW, Charlesworth B, Charlesworth D, & Crow JF (1998). Rates of spontaneous mutation. Genetics, 148(4), 1667–1686. 9560386 10.1093/genetics/148.4.1667 PMC1460098

[45] Cairns J (1975). Mutation selection and the natural history of cancer. Nature, 255(5505), 197–200. 1143315 10.1038/255197a0

[46] Franco I, Helgadottir HT, Moggio A, Larsson M, Vrtacnik P, Johansson A, (2019). Whole genome DNA sequencing provides an atlas of somatic mutagenesis in healthy human cells and identifies a tumor-prone cell type. Genome Biology, 20(1), 285. 31849330 10.1186/s13059-019-1892-z PMC6918713

[47] Martincorena I, & Campbell PJ (2015). Somatic mutation in cancer and normal cells. Science, 349(6255), 1483–1489. 26404825 10.1126/science.aab4082

[48] Moore L, Leongamornlert D, Coorens THH, Sanders MA, Ellis P, Dentro SC, Dawson KJ, Butler T, Rahbari R, Mitchell TJ, Maura F, Nangalia J, Tarpey PS, Brunner SF, Lee-Six H, Hooks Y, Moody S, Mahbubani KT, Jimenez-Linan M, Brosens JJ, Iacobuzio-Donahue CA, Martincorena I, Saeb-Parsy K, Campbell PJ, & Stratton MR (2020). The mutational landscape of normal human endometrial epithelium. Nature, 580(7805), 640–646. 32350471 10.1038/s41586-020-2214-z

[49] Jones S, Zhang X, Parsons DW, Lin JC, Leary RJ, Angenendt P, (2008). Core signaling pathways in human pancreatic cancers revealed by global genomic analyses. Science, 321(5897), 1801–1806. 18772397 10.1126/science.1164368 PMC2848990

[50] Cancer Genome Atlas Research Network. Electronic address, a. a. d. h. e., & Cancer Genome Atlas Research, N. (2017). Integrated genomic characterization of pancreatic ductal adenocarcinoma. Cancer Cell, 32(2), 185–203 e113. 28810144 10.1016/j.ccell.2017.07.007 PMC5964983

[51] Biankin AV, Waddell N, Kassahn KS, Gingras MC, Muthuswamy LB, Johns AL, (2012). Pancreatic cancer genomes reveal aberrations in axon guidance pathway genes. Nature, 491(7424), 399–405. 23103869 10.1038/nature11547 PMC3530898

[52] Waddell N, Pajic M, Patch AM, Chang DK, Kassahn KS, Bailey P, (2015). Whole genomes redefine the mutational landscape of pancreatic cancer. Nature, 518(7540), 495–501. 25719666 10.1038/nature14169 PMC4523082

[53] Kirkwood TB, & Holliday R (1979). The evolution of ageing and longevity. Proceedings of the Royal Society of London - Series B: Biological Sciences, 205(1161), 531–546. 42059 10.1098/rspb.1979.0083

[54] Alberts B, & Sternglanz R (1977). Recent excitement in the DNA replication problem. Nature, 269(5630), 655–661. 201853 10.1038/269655a0

[55] Sung W, Ackerman MS, Miller SF, Doak TG, & Lynch M (2012). Drift-barrier hypothesis and mutation-rate evolution. Proceedings of the National Academy of Sciences of the United States of America, 109(45), 18488–18492. 23077252 10.1073/pnas.1216223109 PMC3494944

[56] Pennycuick A, Teixeira VH, AbdulJabbar K, Raza SEA, Lund T, Akarca AU, Rosenthal R, Kalinke L, Chandrasekharan DP, Pipinikas CP, Lee-Six H, Hynds RE, Gowers KHC, Henry JY, Millar FR, Hagos YB, Denais C, Falzon M, Moore DA, Antoniou S, Durrenberger PF, Furness AJ, Carroll B, Marceaux C, Asselin-Labat ML, Larson W, Betts C, Coussens LM, Thakrar RM, George J, Swanton C, Thirlwell C, Campbell PJ, Marafioti T, Yuan Y, Quezada SA, McGranahan N, & Janes SM (2020). Immune surveillance in clinical regression of pre-invasive squamous cell lung cancer. Cancer Discovery, 10(10), 1489–1499. 32690541 10.1158/2159-8290.CD-19-1366 PMC7611527

[57] Rodriguez RM, Lopez-Vazquez A, & Lopez-Larrea C (2012). Immune systems evolution. Advances in Experimental Medicine and Biology, 739, 237–251. 22399406 10.1007/978-1-4614-1704-0_15

[58] Quach H, Rotival M, Pothlichet J, Loh YE, Dannemann M, Zidane N, (2016). Genetic adaptation and neandertal admixture shaped the immune system of human populations. Cell, 167(3), 643–656. 27768888 10.1016/j.cell.2016.09.024 PMC5075285

[59] Reher D, Key FM, Andres AM, & Kelso J (2019). Immune gene diversity in archaic and present-day humans. Genome Biology and Evolution, 11(1), 232–241. 30566634 10.1093/gbe/evy271 PMC6347564

[60] Marty R, Kaabinejadian S, Rossell D, Slifker MJ, van de Haar J, Engin HB, de Prisco N, Ideker T, Hildebrand WH, Font-Burgada J, & Carter H (2017). MHC-I genotype restricts the oncogenic mutational landscape. Cell, 171(6), 1272–1283. 29107334 10.1016/j.cell.2017.09.050 PMC5711564

[61] Simon AK, Hollander GA, & McMichael A (2015). Evolution of the immune system in humans from infancy to old age. Proceedings of the Biological Sciences, 282(1821), 20143085. 10.1098/rspb.2014.3085 PMC4707740 26702035

[62] Ryan F (1993). The forgotten plague: how the battle against tuberculosis was won—and lost (1st ed.). Boston: Little, Brown.

[63] Greaves M, & Maley CC (2012). Clonal evolution in cancer. Nature, 481(7381), 306–313. 22258609 10.1038/nature10762 PMC3367003

[64] Burrell RA, McGranahan N, Bartek J, & Swanton C (2013). The causes and consequences of genetic heterogeneity in cancer evolution. Nature, 501(7467), 338–345. 24048066 10.1038/nature12625

[65] Polyak K (2014). Tumor heterogeneity confounds and illuminates: a case for Darwinian tumor evolution. Nature Medicine, 20(4), 344–346. 10.1038/nm.3518 24710378

[66] Gatenby RA, & Brown JS (2020). Integrating evolutionary dynamics into cancer therapy. Nature Reviews Clinical Oncology, 17(11), 675–686. 10.1038/s41571-020-0411-1 32699310

[67] Wagle N, Emery C, Berger MF, Davis MJ, Sawyer A, Pochanard P, Kehoe SM, Johannessen CM, MacConaill LE, Hahn WC, Meyerson M, & Garraway LA (2011). Dissecting therapeutic resistance to RAF inhibition in melanoma by tumor genomic profiling. Journal of Clinical Oncology, 29(22), 3085–3096. 21383288 10.1200/JCO.2010.33.2312 PMC3157968

[68] Golan T, Hammel P, Reni M, Van Cutsem E, Macarulla T, Hall MJ, (2019). Maintenance olaparib for germline BRCA-mutated metastatic pancreatic cancer. The New England Journal of Medicine, 381(4), 317–327. 31157963 10.1056/NEJMoa1903387 PMC6810605

[69] Pishvaian MJ, Biankin AV, Bailey P, Chang DK, Laheru D, Wolfgang CL, & Brody JR (2017). BRCA2 secondary mutation-mediated resistance to platinum and PARP inhibitor-based therapy in pancreatic cancer. British Journal of Cancer, 116(8), 1021–1026. 28291774 10.1038/bjc.2017.40 PMC5396101

[70] Yarchoan M, Myzak MC, Johnson BA 3rd, De Jesus-Acosta A, Le DT, Jaffee EM, (2017). Olaparib in combination with irinotecan, cisplatin, and mitomycin C in patients with advanced pancreatic cancer. Oncotarget, 8(27), 44073–44081. 28454122 10.18632/oncotarget.17237 PMC5546463

[71] Barber LJ, Sandhu S, Chen L, Campbell J, Kozarewa I, Fenwick K, Assiotis I, Rodrigues DN, Reis-Filho JS, Moreno V, Mateo J, Molife LR, de Bono J, Kaye S, Lord CJ, & Ashworth A (2013). Secondary mutations in BRCA2 associated with clinical resistance to a PARP inhibitor. The Journal of Pathology, 229(3), 422–429. 23165508 10.1002/path.4140

[72] Sakamoto H, Attiyeh MA, Gerold JM, Makohon-Moore AP, Hayashi A, Hong J, Kappagantula R, Zhang L, Melchor JP, Reiter JG, Heyde A, Bielski CM, Penson AV, Gönen M, Chakravarty D, O’Reilly EM, Wood LD, Hruban RH, Nowak MA, Socci ND, Taylor BS, & Iacobuzio-Donahue CA (2020). The evolutionary origins of recurrent pancreatic cancer. Cancer Discovery, 10(6), 792–805. 32193223 10.1158/2159-8290.CD-19-1508 PMC7323937

[73] Diaz LA Jr., Williams RT, Wu J, Kinde I, Hecht JR, Berlin J, (2012). The molecular evolution of acquired resistance to targeted EGFR blockade in colorectal cancers. Nature, 486(7404), 537–540. 22722843 10.1038/nature11219 PMC3436069

[74] Walther V, Hiley CT, Shibata D, Swanton C, Turner PE, & Maley CC (2015). Can oncology recapitulate paleontology? Lessons from species extinctions. Nature Reviews. Clinical Oncology, 12(5), 273–285. 10.1038/nrclinonc.2015.12 PMC4569005 25687908

[75] Basturk O, Hong SM, Wood LD, Adsay NV, Albores-Saavedra J, Biankin AV, Brosens LA, Fukushima N, Goggins M, Hruban RH, Kato Y, Klimstra DS, Klöppel G, Krasinskas A, Longnecker DS, Matthaei H, Offerhaus GJ, Shimizu M, Takaori K, Terris B, Yachida S, Esposito I, Furukawa T, & Baltimore Consensus Meeting. (2015). A revised classification system and recommendations from the Baltimore consensus meeting for neoplastic precursor lesions in the pancreas. The American Journal of Surgical Pathology, 39(12), 1730–1741. 26559377 10.1097/PAS.0000000000000533 PMC4646710

[76] Kuboki Y, Fischer CG, Beleva Guthrie V, Huang W, Yu J, Chianchiano P, Hosoda W, Zhang H, Zheng L, Shao X, Thompson ED, Waters K, Poling J, He J, Weiss MJ, Wolfgang CL, Goggins MG, Hruban RH, Roberts NJ, Karchin R, & Wood LD (2019). Single-cell sequencing defines genetic heterogeneity in pancreatic cancer precursor lesions. The Journal of Pathology, 247(3), 347–356. 30430578 10.1002/path.5194 PMC6368872

[77] Hata T, Suenaga M, Marchionni L, Macgregor-Das A, Yu J, Shindo K, Tamura K, Hruban RH, & Goggins M (2018). Genome-wide somatic copy number alterations and mutations in high-grade pancreatic intraepithelial neoplasia. The American Journal of Pathology, 188(7), 1723–1733. 29684357 10.1016/j.ajpath.2018.03.012 PMC6024190

[78] Makohon-Moore AP, Matsukuma K, Zhang M, Reiter JG, Gerold JM, Jiao Y, Sikkema L, Attiyeh MA, Yachida S, Sandone C, Hruban RH, Klimstra DS, Papadopoulos N, Nowak MA, Kinzler KW, Vogelstein B, & Iacobuzio-Donahue CA (2018). Precancerous neoplastic cells can move through the pancreatic ductal system. Nature, 561(7722), 201–205. 30177826 10.1038/s41586-018-0481-8 PMC6342205

[79] Iacobuzio-Donahue CA, Velculescu VE, Wolfgang CL, & Hruban RH (2012). Genetic basis of pancreas cancer development and progression: insights from whole-exome and whole-genome sequencing. Clinical Cancer Research, 18(16), 4257–4265. 22896692 10.1158/1078-0432.CCR-12-0315 PMC3422771

[80] Yachida S, Jones S, Bozic I, Antal T, Leary R, Fu B, Kamiyama M, Hruban RH, Eshleman JR, Nowak MA, Velculescu VE, Kinzler KW, Vogelstein B, & Iacobuzio-Donahue CA (2010). Distant metastasis occurs late during the genetic evolution of pancreatic cancer. Nature, 467(7319), 1114–1117. 20981102 10.1038/nature09515 PMC3148940

[81] Hruban RH, Goggins M, Parsons J, & Kern SE (2000). Progression model for pancreatic cancer. Clinical Cancer Research, 6(8), 2969–2972. 10955772

[82] Chen PY, Muzumdar MD, Dorans KJ, Robbins R, Bhutkar A, Del Rosario A, (2018). Adaptive and reversible resistance to Kras inhibition in pancreatic cancer cells. Cancer Research, 78(4), 985–1002. 29279356 10.1158/0008-5472.CAN-17-2129 PMC5837062

[83] Hou P, Kapoor A, Zhang Q, Li J, Wu CJ, Li J, Lan Z, Tang M, Ma X, Ackroyd JJ, Kalluri R, Zhang J, Jiang S, Spring DJ, Wang YA, & DePinho RA (2020). Tumor microenvironment remodeling enables bypass of oncogenic KRAS dependency in pancreatic cancer. Cancer Discovery, 10(7), 1058–1077. 32341020 10.1158/2159-8290.CD-19-0597 PMC7334087

[84] Nagasaka M, Li Y, Sukari A, Ou SI, Al-Hallak MN, & Azmi AS (2020). KRAS G12C Game of Thrones, which direct KRAS inhibitor will claim the iron throne? Cancer Treatment Reviews, 84, 101974. 32014824 10.1016/j.ctrv.2020.101974 PMC7041424

[85] McCormick F (2020). Sticking it to KRAS: covalent inhibitors enter the clinic. Cancer Cell, 37(1), 3–4. 31951561 10.1016/j.ccell.2019.12.009 PMC7891852

[86] Hosoda W, Chianchiano P, Griffin JF, Pittman ME, Brosens LA, Noe M, (2017). Genetic analyses of isolated high-grade pancreatic intraepithelial neoplasia (HG-PanIN) reveal paucity of alterations in TP53 and SMAD4. The Journal of Pathology, 242(1), 16–23. 28188630 10.1002/path.4884 PMC5553451

[87] Noe M, Niknafs N, Fischer CG, Hackeng WM, Beleva Guthrie V, Hosoda W, (2020). Genomic characterization of malignant progression in neoplastic pancreatic cysts. Nature Communications, 11(1), 4085. 10.1038/s41467-020-17917-8 PMC7428044 32796935

[88] Fujikura K, Hosoda W, Felsenstein M, Song Q, Reiter JG, Zheng L, Beleva Guthrie V, Rincon N, Dal Molin M, Dudley J, Cohen JD, Wang P, Fischer CG, Braxton AM, Noë M, Jongepier M, Fernández-del Castillo C, Mino-Kenudson M, Schmidt CM, Yip-Schneider MT, Lawlor RT, Salvia R, Roberts NJ, Thompson ED, Karchin R, Lennon AM, Jiao Y, & Wood LD (2020). Multiregion whole-exome sequencing of intraductal papillary mucinous neoplasms reveals frequent somatic KLF4 mutations predominantly in low-grade regions. Gut, gutjnl-2020–321217. 10.1136/gutjnl-2020-321217 PMC8262510 33028669

[89] Tarabichi M, Martincorena I, Gerstung M, Leroi AM, Markowetz F, Evolution P, (2018). Neutral tumor evolution? Nature Genetics, 50(12), 1630–1633. 30374075 10.1038/s41588-018-0258-x PMC6548558

[90] Williams MJ, Werner B, Barnes CP, Graham TA, & Sottoriva A (2016). Identification of neutral tumor evolution across cancer types. Nature Genetics, 48(3), 238–244. 26780609 10.1038/ng.3489 PMC4934603

[91] Durrett R (2013). Population genetics of neutral mutations in exponentially growing cancer cell populations. The Annals of Applied Probability, 23(1), 230–250. 23471293 10.1214/11-aap824 PMC3588108

[92] Martincorena I, Raine KM, Gerstung M, Dawson KJ, Haase K, Van Loo P, (2017). Universal patterns of selection in cancer and somatic tissues. Cell, 171(5), 1029–1041. 29056346 10.1016/j.cell.2017.09.042 PMC5720395

[93] Yoshida K, Gowers KHC, Lee-Six H, Chandrasekharan DP, Coorens T, Maughan EF, Beal K, Menzies A, Millar FR, Anderson E, Clarke SE, Pennycuick A, Thakrar RM, Butler CR, Kakiuchi N, Hirano T, Hynds RE, Stratton MR, Martincorena I, Janes SM, & Campbell PJ (2020). Tobacco smoking and somatic mutations in human bronchial epithelium. Nature, 578(7794), 266–272. 31996850 10.1038/s41586-020-1961-1 PMC7021511

[94] Lee-Six H, Olafsson S, Ellis P, Osborne RJ, Sanders MA, Moore L, Georgakopoulos N, Torrente F, Noorani A, Goddard M, Robinson P, Coorens THH, O’Neill L, Alder C, Wang J, Fitzgerald RC, Zilbauer M, Coleman N, Saeb-Parsy K, Martincorena I, Campbell PJ, & Stratton MR (2019). The landscape of somatic mutation in normal colorectal epithelial cells. Nature, 574(7779), 532–537. 31645730 10.1038/s41586-019-1672-7

[95] Priestley P, Baber J, Lolkema MP, Steeghs N, de Bruijn E, Shale C, Duyvesteyn K, Haidari S, van Hoeck A, Onstenk W, Roepman P, Voda M, Bloemendal HJ, Tjan-Heijnen VCG, van Herpen CML, Labots M, Witteveen PO, Smit EF, Sleijfer S, Voest EE, & Cuppen E (2019). Pan-cancer whole-genome analyses of metastatic solid tumours. Nature, 575(7781), 210–216. 31645765 10.1038/s41586-019-1689-y PMC6872491

[96] Ryser MD, Mallo D, Hall A, Hardman T, King LM, Tatishchev S, Sorribes IC, Maley CC, Marks JR, Hwang ES, & Shibata D (2020). Minimal barriers to invasion during human colorectal tumor growth. Nature Communications, 11(1), 1280. 10.1038/s41467-020-14908-7 PMC7062901 32152322

[97] Adsay V, Logani S, Sarkar F, Crissman J, & Vaitkevicius V (2000). Foamy gland pattern of pancreatic ductal adenocarcinoma: a deceptively benign-appearing variant. The American Journal of Surgical Pathology, 24(4), 493–504. 10757396 10.1097/00000478-200004000-00003

[98] Kanai N, Nagaki S, & Tanaka T (1987). Clear cell carcinoma of the pancreas. Acta Pathol Jpn, 37(9), 1521–1526. 3687432 10.1111/j.1440-1827.1987.tb02273.x

[99] Jiang X, & Tomlinson IPM (2020). Why is cancer not more common? A changing microenvironment may help to explain why, and suggests strategies for anti-cancer therapy. Open Biology, 10(4), 190297. 32289242 10.1098/rsob.190297 PMC7241076

[100] Olive KP, Jacobetz MA, Davidson CJ, Gopinathan A, McIntyre D, Honess D, Madhu B, Goldgraben MA, Caldwell ME, Allard D, Frese KK, DeNicola G, Feig C, Combs C, Winter SP, Ireland-Zecchini H, Reichelt S, Howat WJ, Chang A, Dhara M, Wang L, Ruckert F, Grutzmann R, Pilarsky C, Izeradjene K, Hingorani SR, Huang P, Davies SE, Plunkett W, Egorin M, Hruban RH, Whitebread N, McGovern K, Adams J, Iacobuzio-Donahue C, Griffiths J, & Tuveson DA (2009). Inhibition of Hedgehog signaling enhances delivery of chemotherapy in a mouse model of pancreatic cancer. Science, 324(5933), 1457–1461. 19460966 10.1126/science.1171362 PMC2998180

[101] Whittle MC, & Hingorani SR (2019). Fibroblasts in pancreatic ductal adenocarcinoma: biological mechanisms and therapeutic targets. Gastroenterology, 156(7), 2085–2096. 30721663 10.1053/j.gastro.2018.12.044 PMC6486863

[102] Neesse A, Algul H, Tuveson DA, & Gress TM (2015). Stromal biology and therapy in pancreatic cancer: a changing paradigm. Gut, 64(9), 1476–1484. 25994217 10.1136/gutjnl-2015-309304

[103] Fujikura K, Hutchings D, Braxton AM, Zhu Q, Laheru DA, Hruban RH, Thompson ED, & Wood LD (2020). Intraductal pancreatic cancer is less responsive than cancer in the stroma to neoadjuvant chemotherapy. Modern Pathology, 33(10), 2026–2034. 32457408 10.1038/s41379-020-0572-6 PMC7529702

[104] Geistlinger L, Oh S, Ramos M, Schiffer L, LaRue RS, Henzler CM, Munro SA, Daughters C, Nelson AC, Winterhoff BJ, Chang Z, Talukdar S, Shetty M, Mullany SA, Morgan M, Parmigiani G, Birrer MJ, Qin LX, Riester M, Starr TK, & Waldron L (2020). Multi-omic analysis of subtype evolution and heterogeneity in high-grade serous ovarian carcinoma. Cancer Research, 80, 4335–4345. 32747365 10.1158/0008-5472.CAN-20-0521 PMC7572645

[105] Warren JT, & Link DC (2020). Clonal hematopoiesis and risk for hematologic malignancy. Blood, 136(14), 1599–1605. 32736382 10.1182/blood.2019000991 PMC8209630

[106] Shibata D (2020). Visualizing human colorectal cancer intratumor heterogeneity with phylogeography. iScience, 23(7), 101304. 32623333 10.1016/j.isci.2020.101304 PMC7334596

[107] Hruban RH, & Lillemoe KD (2019). Screening for pancreatic cancer gets a D, but the student is improving. JAMA Surgery, 154(9), 795–797. 31386099 10.1001/jamasurg.2019.2832

[108] Andea A, Sarkar F, & Adsay VN (2003). Clinicopathological correlates of pancreatic intraepithelial neoplasia: a comparative analysis of 82 cases with and 152 cases without pancreatic ductal adenocarcinoma. Modern Pathology, 16(10), 996–1006. 14559982 10.1097/01.MP.0000087422.24733.62

[109] Laffan TA, Horton KM, Klein AP, Berlanstein B, Siegelman SS, Kawamoto S, Johnson PT, Fishman EK, & Hruban RH (2008). Prevalence of unsuspected pancreatic cysts on MDCT. AJR. American Journal of Roentgenology, 191(3), 802–807. 18716113 10.2214/AJR.07.3340 PMC2692243

[110] Oyama H, Tada M, Takagi K, Tateishi K, Hamada T, Nakai Y, Hakuta R, Ijichi H, Ishigaki K, Kanai S, Kogure H, Mizuno S, Saito K, Saito T, Sato T, Suzuki T, Takahara N, Morishita Y, Arita J, Hasegawa K, Tanaka M, Fukayama M, & Koike K (2020). Long-term risk of malignancy in branch-duct intraductal papillary mucinous neoplasms. Gastroenterology, 158(1), 226–237. 31473224 10.1053/j.gastro.2019.08.032

[111] Srivastava S, Koay EJ, Borowsky AD, De Marzo AM, Ghosh S, Wagner PD, (2019). Cancer overdiagnosis: a biological challenge and clinical dilemma. Nature Reviews. Cancer, 19(6), 349–358. 31024081 10.1038/s41568-019-0142-8 PMC8819710

[112] Izumo W, Higuchi R, Furukawa T, Yazawa T, Uemura S, Shiihara M, & Yamamoto M (2020). Importance of each high-risk stigmata and worrisome features as a predictor of high-grade dysplasia in intraductal papillary mucinous neoplasms of the pancreas. Pancreatology, 20(5), 895–901. 32624417 10.1016/j.pan.2020.06.011

[113] Sharib JM, Fonseca AL, Swords DS, Jaradeh K, Bracci PM, Firpo MA, Hatcher S, Scaife CL, Wang H, Kim GE, Mulvihill SJ, Maitra A, Koay EJ, & Kirkwood KS (2018). Surgical overtreatment of pancreatic intraductal papillary mucinous neoplasms: do the 2017 International Consensus Guidelines improve clinical decision making? Surgery, 164(6), 1178–1184. 30170819 10.1016/j.surg.2018.07.014

[114] Fischer CG, Beleva Guthrie V, Braxton AM, Zheng L, Wang P, Song Q, Griffin JF, Chianchiano PE, Hosoda W, Niknafs N, Springer S, Dal Molin M, Masica D, Scharpf RB, Thompson ED, He J, Wolfgang CL, Hruban RH, Roberts NJ, Lennon AM, Jiao Y, Karchin R, & Wood LD (2019). Intraductal papillary mucinous neoplasms arise from multiple independent clones, each with distinct mutations. Gastroenterology, 157(4), 1123–1137. 31175866 10.1053/j.gastro.2019.06.001 PMC6756950

[115] Felsenstein M, Noe M, Masica DL, Hosoda W, Chianchiano P, Fischer CG, (2018). IPMNs with cooccurring invasive cancers: neighbours but not always relatives. Gut, 67(9), 1652–1662. 29500184 10.1136/gutjnl-2017-315062 PMC10489026

[116] Terhune PG, Phifer DM, Tosteson TD, & Longnecker DS (1998). K-ras mutation in focal proliferative lesions of human pancreas. Cancer Epidemiology, Biomarkers & Prevention, 7(6), 515–521. 9641496

[117] Han Y, Lee H, Kang JS, Kim JR, Kim HS, Lee JM, Lee KB, Kwon W, Kim SW, & Jang JY (2018). Progression of pancreatic branch duct intraductal papillary mucinous neoplasm associates with cyst size. Gastroenterology, 154(3), 576–584. 29074452 10.1053/j.gastro.2017.10.013

[118] Choi SH, Park SH, Kim KW, Lee JY, & Lee SS (2017). Progression of unresected intraductal papillary mucinous neoplasms of the pancreas to cancer: a systematic review and meta-analysis. Clinical Gastroenterology and Hepatology, 15(10), 1509–1520. 28342950 10.1016/j.cgh.2017.03.020

[119] Kuboki Y, Fischer CG, Beleva Guthrie V, Huang W, Yu J, Chianchiano P, (2018). Single-cell sequencing defines genetic heterogeneity in pancreatic cancer precursor lesions. The Journal of Pathology, 247(3), 347–356. 10.1002/path.5194 PMC6368872 30430578

[120] Davies L, Ouellette M, Hunter M, & Welch HG (2010). The increasing incidence of small thyroid cancers: where are the cases coming from? Laryngoscope, 120(12), 2446–2451. 21108428 10.1002/lary.21076

[121] Ahn HS, Kim HJ, & Welch HG (2014). Korea’s thyroid-cancer “epidemic”—screening and overdiagnosis. The New England Journal of Medicine, 371(19), 1765–1767. 25372084 10.1056/NEJMp1409841

[122] Blackford AL, Canto MI, Klein AP, Hruban RH, & Goggins M (2020). Recent trends in the incidence and survival of stage 1A pancreatic cancer: a surveillance, epidemiology, and end results analysis. Journal of the National Cancer Institute(epub ahead of print), 112, 1162–1169. 31958122 10.1093/jnci/djaa004 PMC7669234

